# CPAP and high-flow nasal oxygen also reduce lung, diaphragm, and accessory muscle injury in experimental self-inflicted lung injury

**DOI:** 10.1038/s41598-026-39564-7

**Published:** 2026-02-11

**Authors:** Sonia Reveco, Felipe M. Llancalahuen, Paola Caviedes, Andrés Silva, Javier Contreras, Carlos González, Benjamín Erranz, Agustín Pérez, Juan P. Cruces, Daniel E. Hurtado, Pablo Cruces

**Affiliations:** 1 Unidad de Paciente Crítico Pediátrico, Hospital El Carmen Dr. Luis Valentín Ferrada, Santiago, Chile; 2 Laboratory of Translational Research in Critical Care (LTRCC), Departamento de Medicina Intensiva, Facultad de Medicina, Pontificia Universidad Católica de Chile, Santiago, Chile; 3 Laboratorio de Genética y Patogénesis Bacteriana, Centro de Investigación de Resiliencia a Pandemias, Facultad de Ciencias de la Vida, Universidad Andres Bello, Santiago, Chile; 4 Laboratorio integrativo de Biomecánica y Fisiología del Esfuerzo (LIBFE), Escuela de Kinesiología, Universidad de los Andes, Santiago, Chile; 5 School of Veterinary Medicine, One Health Institute, Faculty of Life Sciences, Universidad Andres Bello, Santiago, Chile; 6 Department of Structural and Geotechnical Engineering, School of Engineering, Pontificia Universidad Católica de Chile, Santiago, Chile; 7 Institute for Biological and Medical Engineering, Schools of Engineering, Medicine and Biological Sciences, Pontificia Universidad Católica de Chile, Santiago, Chile; 8 Laboratory of Translational Research in Critical Care, Center for Research on Pandemic Resilience, Faculty of Life Sciences, Universidad Andres Bello, Santiago, Chile

**Keywords:** Acute lung injury, Continuous positive airway pressure, High-flow nasal oxygen, Spontaneous breathing, Respiratory effort, Underassistance myotrauma, Patient self-inflicted lung injury, Diseases, Health care, Medical research, Physiology

## Abstract

Strenuous respiratory effort has been proposed as a second hit, introducing the concepts of *“patient self-inflicted lung injury”* (P-SILI) and *underassistance myotrauma*. We investigated the effects of continuous positive airway pressure (CPAP) and high-flow nasal oxygen (HFNO) on inspiratory and expiratory effort, lung injury, and myotrauma of obligatory and accessory respiratory muscles in experimental P-SILI. In rats, lung injury was induced through surfactant depletion, followed by 3 h of standard oxygen therapy (2 L/min, positive control) or supported ventilation (CPAP 6 cmH2O, HFNO 4 L/min, and protective mechanical ventilation [MV, negative control] groups). Subjects were assessed through esophageal manometry, surface electromyography on the abdominal wall, thoracic ultrasound, plasma multiplex analysis, and histology on the lungs, diaphragm, and accessory inspiratory and expiratory muscles. Compared against standard oxygen therapy, the MV and CPAP groups resulted in lower inspiratory and expiratory efforts and diaphragm excursion. Furthermore, intragroup analysis showed that CPAP also reduced respiratory rate and prevented the loss of lung aeration. All support therapies prevented lung, diaphragm, and accessory muscle injuries, but with nuances. In lungs and accessory muscles, MV showed the highest injury protection and HFNO the lowest, while CPAP was the most protective for the diaphragm. Only MV resulted in a lower plasma GRO-α concentration than standard oxygen therapy. Invasive and non-invasive respiratory support therapies are effective in protecting the lungs and respiratory muscles, but the effect is stepped in the lung and varies between obligatory and accessory respiratory muscles.

**Supplementary Information:**

The online version contains supplementary material available at 10.1038/s41598-026-39564-7.

## Introduction

Preclinical research suggests that strenuous respiratory effort contributes to the progression of both pulmonary and diaphragmatic injury in subjects with acutely and severely injured lungs, a phenomenon termed patient-self-inflicted lung injury (P-SILI) and *underassistance myotrauma*^1–6^. This excessive respiratory effort increases tissue damage and regional strain, as well as the strain rate across the different structures of the respiratory system^7,8^. Conversely, the progression of lung injury and associated mechanobiological phenomena can be attenuated or prevented by early use of protective mechanical ventilation (MV)^3,9,10^.

In the clinical setting, there is a growing interest in preventing MV and its associated complications. Noninvasive support therapies such as continuous positive airway pressure (CPAP) and high-flow nasal oxygen (HFNO) have emerged as promising alternatives, aiming to improve oxygenation and reduce respiratory effort^4,11–21^. However, their protective effects remain understudied, both within the pulmonary parenchyma and across the obligatory (diaphragm) and accessory inspiratory and expiratory muscles. We hypothesized that noninvasive support therapies reduce both, as assessed by compensatory respiratory symptoms, gas exchange, inspiratory effort, work of breathing, expiratory effort, diaphragm excursion, lung aeration loss, plasma biomarkers, and lung, obligatory, and accessory respiratory muscles histology in a preclinical P-SILI model.

## Methods

### Sample size

Based on a previous murine P-SILI study with six subjects per group, we performed a power analysis using G*Power 3 software (Version 3.1.9.6, Heinrich-Heine-Universität Düsseldorf). We determined the sample size considering data from morphological analysis, specifically from differences between the semi-quantitative lung injury score in the Unassisted and MV groups^9^. An effect size of 1.31 was determined from the difference in means **±** SD between the Unassisted and MV groups (5.22 ± 1.96 vs. 2.78 ± 1.75, respectively). Power was calculated using the nonparametric Wilcoxon–Mann–Whitney test, with an alpha error of 0.05 and a power of 0.9, resulting in a sample size of 8 animals per group. Projecting a mortality of 33%, we adjust to 11 subjects per group.

### Animal preparation

The Pontificia Universidad Católica de Chile Bioethics Committee approved the study protocol (Approval Act ID 230411006, November 2024). Fifty-five balanced by sex, specific pathogen-free Sprague-Dawley rats were used in this study (Center for Innovation in Biomedical Experimental Models, CIBEM, Pontificia Universidad Católica de Chile). The rats were maintained in a humidity, light, and temperature-controlled environment inside a dedicated animal research facility. Food and water were provided *ad libitum*.

### Anesthesia

The subjects were placed in an Anesthesia Induction Chamber (Harvard Apparatus, MA, USA) connected to an Anesthetic Vaporizer (Harvard Apparatus, MA, USA), starting with pre-oxygenation (inspiratory fraction of oxygen, F_I_O_2_ = 100%) followed by an inhalational induction of 3% isoflurane. Anesthesia adequacy was assessed sequentially by the absence of the pedal reflex. Next, the animals were temporarily removed from the induction chamber, and intraperitoneal maintenance anesthesia consisting of ketamine (30 mg·kg^− 1^) and xylazine (5 mg·kg^− 1^) was administered, immediately returning to the chamber until the anesthetic adequacy was confirmed. Subsequently, they were set in the intubation stand in Fowler’s position, maintaining inhalational anesthesia and preoxygenation through a Low-Profile Nasal Anesthesia Mask (VetFlo™, Kent Scientific, USA).

### Intubation and acute lung injury

Firstly, videolaringoscopy was performed (Bebird R3 system, Heifeng Zhizao Technology Co, Shenzhen, Guangdong, China). Then, a laryngeal instillation of 2% lidocaine (10 µL) through a micropipette was administered, and tracheal intubation was performed using a 16G BD Angiocath^®^ catheter (Becton, Dickinson, Utah, USA).

Animals were supported during 5 min in volume-controlled ventilation with the following settings: Vt of 6 ml kg^− 1^, positive end-expiratory pressure (PEEP) of 5 cmH_2_O, I:E ratio of 1:2, respiratory rate (RR) of 90/min^− 1^, and a F_I_O_2_ of 0.6 (VentElite^®^ Small Animal Ventilator, Harvard Apparatus, MA, USA). Next, acute lung injury (ALI) was induced by surfactant depletion and lung collapse. Briefly, 5 ml·kg^− 1^ of warm normal saline were flushed into the trachea, followed by suctioning of the residual fluid from the airway^9^. In previous studies, we have used this method to induce moderate to severe hypoxemia in spontaneously breathing subjects, with an acceptable mortality rate, thereby allowing us to characterize lung and diaphragmatic injury added by the respiratory effort itself^4,8–10^. After lavage, the animals were positioned supine and stabilized in MV for 10 min.

### Esophageal catheter placement

To ensure the correct esophageal positioning and comfort, we used a soft double-lumen 4.5 Fr water-filled catheter of 6 cm in length, with a guidewire placed in the distal lumen only to facilitate the insertion (Multicath2, Vygon, Germany). The proximal lumen was connected to a differential pressure transducer (FE141, ADInstrument^®^, Dunedin, New Zealand) for esophageal manometry, calibrated at 0 and 10 mmHg. The appropriate position of the esophageal catheter was tested by the presence of the cardiac artifact on the esophageal pressure tracing and confirmed by the oscillations during a rapid, manual rib cage compression similar to an “occlusion test”^22^.

### Experimental groups

Block randomization was used to assign the animals to the following groups: lung injury plus controlled MV (MV, negative control), lung injury plus standard low-flow oxygen therapy (Unassisted, positive control), lung injury plus CPAP, lung injury plus HFNO, and non-injured (Sham).

The MV group maintained the same ventilatory setting, which was lung protective in previous rodent studies^9,10^. Except for the MV group, weaning from MV was performed following stabilization and detection of spontaneous respiratory effort, and all animals were kept in FIO_2_ of 60%.

The CPAP group was placed on a continuous positive airway pressure device (ResMed H5i^®^, San Diego, USA) connected to a Full-Face Anesthesia Mask (VetFlo™, Kent Scientific, USA) to maintain a constant pressure of 6 cmH_2_O, which was also protective for lungs and diaphragm in previous rodent studies^4,8^.

The HFNO group was placed on a specific device (Airvo™ 2 Nasal High Flow/HFNO System, Fisher & Paykel Healthcare, Auckland, New Zealand) connected to the Low-Profile Nasal Anesthesia Mask to maintain a constant flow of humidified 4 L/min, heated at 37 °C. This flow was close to 12 ml/min/kg, which is a high flow rate for extremely preterm infants weighing less than 1000 g^23^.

The Unassisted and Sham groups were connected to standard oxygen therapy using a continuous flow of 2 L/min through a Low-Profile Nasal Anesthesia Mask.

The animals were observed for 3 h. Ketamine (15 mg·kg^− 1^) and xylazine (2.5 mg·kg^− 1^) were administered every hour. The pedal reflex was monitored every 15 min. In cases where the reflex persisted, inhalational anesthesia was briefly restarted at 1% isoflurane. This strategy enabled adequate sedation and spontaneous respiratory effort in previous studies^4,8–10^.

### General monitoring

Measurements were registered at baseline, which was established twenty minutes after stabilization, at the beginning [T0], middle [T1.5], and the end of the observation period [T3]. At each time point, rectal temperature (37 °C), heart rate, RR, and peripheral oxygen saturation (SpO_2_) were monitored using the Small Animal Physiological Monitoring System (Harvard Apparatus, MA, USA).

Also, we qualitatively evaluated compensatory respiratory symptoms: nasal flaring, sternocleidomastoid, and abdominal muscle use. The variables received a score between 0 (absence) and 3 (maximum intensity): Nasal flaring was visually determined by noticing widening of the nostrils during inspiration, sternocleidomastoid muscle use was determined by gentle palpation of its clavicular insertion during inspiration, and abdominal muscle use was determined by gentle palpation of the abdomen during expiration^8^.

Gas exchange: at T0 and T3, 100µL of blood samples were collected from the second third of the lower tail artery using a 27G 13 mm needle (Nipro, Bridgewater, USA) connected to a 1 mL arterial blood collection BD A-Line™ syringe. Samples were assessed using a point-of-care blood analyzer (BGEM Test Cards, Epocal Inc., Siemens Healthcare Manufacturing Ltd, Dublin, Ireland).

### Advanced monitoring

We sequentially performed esophageal manometry, lung and diaphragm ultrasound (US), and surface electromyography (sEMG) in the rectus abdominis muscle at T0 and T3.

Esophageal manometry: inspiratory effort was assessed as negative esophageal pressure swings (DPes). The maximal negative deflection of the esophageal pressure was defined as ΔPes, calculated over a mean of 30 s (more than 20 breaths)^24,25^. Next, the pressure rate product per minute (PRP = ΔPes x RR) was assessed as a surrogate of work of breathing (WOB). In the absence of respiratory effort (controlled MV), we recorded a ΔPes = 0 [see Additional Figure S1].

Diaphragm US: We evaluated diaphragm excursion in the right hemithorax, which reflects the amplitude of the active movement of the diaphragm during the respiratory cycle. In the MV group, we recorded the passive excursion. The probe was placed at the junction of the anterior and posterior axillary lines and the lower edge of the right costal arch. The liver was used as the acoustic window for the diaphragm. The probe was pointed to the head and back, and diaphragm excursion, inspiratory time, and expiratory time were displayed under M-mode [see Additional Figure S2].

Lung transthoracic US: Images were obtained using B-mode. Each lung was divided into four regions: anterior right, lateral right, anterior left, and lateral left. For each zone, a score of 0–3 was assigned according to the observed pattern. The four US patterns and the scores given for each were as follows: (1) normal pattern, presence of lung sliding and artifactual horizontal A-lines (0 points); (2) B-pattern, presence of two or more well-defined vertical B-lines extending from the pleural line (1 point); (3) severe B- pattern, multiple confluent vertical B-lines extending from the pleural line (2 points); and (4) lung consolidation, presence of tissue structure with or without hyperechoic punctiform images resembling air bronchograms (3 points)^26,27^. The lung US score was assessed by adding the worst finding in each region of interest, ranging from 0 to 12 points. Lung US score quantified the loss of lung aeration in real time [see Additional Figure S3]. For all ultrasound assessments, depth was set to 3 cm, and the broadband linear array probe was set at 19 MHz (Sonosite PX Stand Series, Fujifilm Sonosite Inc., Seattle, USA). The mean of two valid measurements was recorded, defined as those with a < 10% difference. All the US images were analyzed offline in a blind manner.

Surface electromyography (sEMG): It was performed on the abdominal wall to quantify expiratory effort. The animals’ skin was prepared by removing their fur from the abdomen and then cleaned with alcohol. EMG signals were acquired by placing electrodes 2 cm apart, using an eight-channel bioamplifier, and converted to digital. These signals were recorded at 2000 Hz by a data acquisition software (Trigno^®^ Avanti System; Delsys, Greater Manchester, UK)^28^. Raw EMG signals were processed through notch and band-pass filters to eliminate artifact noise (Chebyshev type II and 20–500 Hz filters, respectively). For spectral characterization, the first 5 min of filtered data were analyzed. Power spectral density was computed via Welch’s method, expressed as mean frequency in Hertz (Hz). Critically, while the time-domain EMG amplitude reflects microvolt measurements, frequency-domain transformations represent signal components, where spectral amplitudes are proportional to the original voltage due to normalization and windowing effects. This approach enables precise quantification of physiological bandwidths and noise profiles, including harmonics (e.g., 50/60 Hz interference) and myoelectric signatures, assessing the accessory expiratory muscle load [see Additional Figure S4]. EMG signals were also analyzed offline in a blind manner.

### Euthanasia

Euthanasia was performed by a lethal intraperitoneal dose of thiopental (200 mg/kg) and a cardiac exsanguination after deepening inhalational anesthesia with 5% isoflurane (deep surgical plane). Blood samples were collected using a PrecisionGlide™ G21 through a BD Vacutainer^®^ tube with lithium heparin. The samples were centrifuged at 3000 rpm for 5 min to obtain plasma, which was aliquoted and stored at -80 °C for later measurements.

### Plasma biomarkers

Multiplex analysis for quantification of inflammatory (IL-1β and TNF-α) and immunomodulatory (IL-2) cytokines, chemokine (GRO-α), and soluble endothelial adhesion molecules (ICAM-1 and VCAM-1) in plasma was performed using Luminex 200^®^ (Luminex Corporation, Austin, USA). Lactate dehydrogenase and total creatine kinase were assessed by colorimetric activity test and MiniChemi^®^ Biochemistry Analyzer, respectively.

### Lungs and respiratory muscles histology

After confirming the absence of vital signs, a bolus of 4% formaldehyde and 0.5% glutaraldehyde in 0.2 M HEPES buffer (pH 7.4) was introduced in a liquid column into the airway until a pressure of 25 cmH_2_O was reached, fixing the lung tissue.

Subsequently, the chest cavity was opened, and the lungs and respiratory muscles were removed and classified as: (1) Inspiratory obligatory muscle: diaphragm; (2) Intrathoracic inspiratory accessory muscle: external intercostals; (3) Extrathoracic inspiratory accessory muscle: sternocleidomastoid; (4) Intrathoracic expiratory accessory muscle: internal intercostals; and (5) Extrathoracic expiratory accessory muscle: rectus abdominis. Lungs and muscles were fixed using the same fixative solution. The right lungs were cross-sectioned every 1 mm from apex to base (22–24 slices per lung), with analysis of only the twentieth lung slice, due to a homogeneous distribution of lung damage from apical to basal regions reported in a previous murine P-SILI study^10^. For each section, the median of ten random fields, at 200x magnification, was reported. Image-Pro Plus was used as image analysis software (ver. 7.0.1.658, Media Cybernetics, Rockville, MD, USA).

We used a multiparametric and semiquantitative lung injury score to characterize the predominant structural damage. It includes thirteen parameters, grouped into five sub-categories: airway and alveolar epithelial injuries, vascular injury, inflammatory and fibroproliferative response, scored as 0 (absent), 1 (mild), 2 (moderate), and 3 (severe)^4,10^. We used an aggregate injury score, consisting of the sum of the scores of the individual lung injury parameters, which represents the severity of lung injury.

Through a similar semiquantitative approach, we evaluated vascular changes (edema, hyperemia, microhemorrhage, and thrombosis), muscle fiber injury (swelling, fragmentation, mispositioned myonuclei, and condensation), intensity of inflammation, and fibrosis in the respiratory muscles. Mispositioned myonuclei and condensation are markers of muscle dysfunction, and degeneration and regeneration, respectively^29^. We also used an aggregate muscle injury score.

Muscle fiber necrosis was also assessed in the diaphragm. A board-certified pathologist (CG), blind to experimental groups, analyzed the samples.

A schematic depicting the experimental protocol can be found in Additional Figure S5.

### Statistical analysis

Normality of the data was evaluated using the Shapiro–Wilk test. For intergroup comparisons at each time point, Welch’s t-test was applied when all groups satisfied normality; otherwise, the Mann–Whitney U-test was used. Benjamini–Hochberg comparison correction was applied to control for a False Discovery Rate. Intragroup differences between T0 and T3 were assessed using the Wilcoxon signed-rank test. We calculated the nonparametric Spearman’s correlation (rho) between absolute values of inspiratory effort, WOB, and expiratory effort with the aggregated lung, diaphragm, and accessory muscle injury scores. To determine the strength of the association, the absolute values of rho were classified as weak 0.2–0.39, moderate 0.40–0.59, and strong *≥* 0.6. Data were presented as median (IQR). Significance was set at *p* < 0.05. All statistical analyses were performed in Python using SciPy v1.11.1.

## Results

Out of 55 animals, four died immediately after saline lavage, before assignment to an experimental group. The remaining 51 were divided into 4 groups of 10 (MV, CPAP, Unassisted, and Sham) and 1 group of 11 (HFNO). During the experimental protocol, mortality occurred in 3 subjects of the Unassisted group and 2 of the HFNO group, all of them during the last hour of observation. There was no mortality in the other groups.

At baseline, there were no differences in weight, sex, and RR between the MV, CPAP, HFNO, and Unassisted groups. Surfactant depletion resulted in an SpO_2_ lower than 88% in all the groups. An additional file shows the physiological data for the experimental groups at baseline in more detail [see Additional Table S1].

### General monitoring

During the observation period, the MV group exhibited lower compensatory symptoms (nasal flaring, inspiratory and expiratory muscle activation) than the CPAP, HFNO, and Unassisted groups. The CPAP group exhibited lower nasal flaring and expiratory muscle activation than the Unassisted and HFNO groups at the end of the study (all *p* < 0.05). There were no differences in oxygenation between the injured groups during the overall experimental period, but the MV group tended to have higher oxygenation than the CPAP, HFNO, and Unassisted groups at the end of the study. Additionally, the MV and CPAP groups had lower PaCO_2_ than the Unassisted and HFNO groups at T0 and T3 (*p* < 0.05).

Regarding the lung US score, the MV group had a lower score than the CPAP, HFNO, and Unassisted groups at T0. The MV and CPAP groups also presented a lower score than HFNO at T3 (both *p* < 0.05).

Intragroup analysis showed that the CPAP group exhibited a reduction of RR, nasal flaring, expiratory muscle activation, lung US score, and prolonged expiratory time at T3 (all *p* < 0.05) (Table 1).

**Table 1:** Physiological data, respiratory times, and lung ultrasound score for the experimental groups during the observation period.

	Unassisted	HFNO	CPAP	MV	Sham
Respiratory rate (breaths per minute)					
T0	100.0 (35.0)	76.0 (54.0)	94.5 (8.8)	90.0 (0.0)	46.0 (24.5)*†
Middle	96.0 (15.5)	72.0 (64.0)	70.0 (15.0)*†§	90.0 (0.0)	62.5 (16.8)*†
T3	82.0 (20.5)	72.0 (30.0)†	76.5 (10.2)†§	90.0 (0.0)	60.0 (14.5)*†
Nasal flaring (score)					
T0	1.0 (0.5)†	1.0 (0.0)†	1.5 (1.0)†	0.0 (0.0)*	0.0 (0.0)*
Middle	2.0 (1.0)†	1.0 (1.0)†	1.0 (0.0)*†	0.0 (0.0)*	0.0 (0.0)*
T3	2.0 (1.0)†	2.0 (1.0)†‡§	1.0 (0.0)*†‡	0.0 (0.0)*	0.0 (0.0)*
Inspiratory muscle activation (score)					
T0	1.0 (0.0)†	1.0 (0.0)†	1.0 (0.0)†	0.0 (0.0)*	0.0 (0.0)*
Middle	1.0 (0.5)†	1.0 (0.0)†	1.0 (0.0)†	0.0 (0.0)*	0.0 (0.0)*
T3	1.0 (0.5)†	1.0 (0.0)†	1.0 (0.0)†	0.0 (0.0)*	0.0 (0.0)*
Expiratory muscle activation (score)					
T0	2.0 (0.0)†	2.0 (0.0)†	2.0 (0.0)†	0.0 (0.0)*	0.0 (0.0)*
Middle	2.0 (0.5)†	2.0 (1.0)†‡	2.0 (1.0)*†‡	0.0 (0.0)*	0.0 (0.0)*
T3	2.0 (0.5)†	2.0 (1.0)†‡	1.0 (0.8)*†‡§	0.0 (0.0)*	0.0 (0.0)*
PaO_2_ (mmHg)					
T0	84.8 (26.7)	97.8 (82.8)	94.2 (44.7)	94.7 (134.7)	182.9 (83.5)*
T3	91.5 (31.8)	123.0 (63.8)	92.5 (44.2)	171.6 (159.0)	176.8 (50.6)*
PaCO_2_ (mmHg)					
T0	66.6 (21.7)	76.6 (30.0)†‡	46.4 (9.0)*‡	48.1 (22.0)	44.3 (14.8)*
T3	61.2 (15.2)	76.2 (31.5)†‡	48.5 (7.0)*‡	47.2 (13.0)	47.1 (10.9)*
Inspiratory time (s)					
T0	0.2 (0.1)	0.2 (0.1)	0.2 (0.1)	0.2 (0.0)	0.3 (0.1)*†
T3	0.2 (0.1)	0.2 (0.0)	0.2 (0.1)	0.2 (0.0)	0.3 (0.1)†
Expiratory time (s)					
T0	0.4 (0.2)	0.6 (0.3)	0.5 (0.1)	0.4 (0.0)	1.0 (0.8)*†
T3	0.5 (0.2)	0.6 (0.3)	0.6 (0.1)†§	0.4 (0.0)	0.7 (0.3)*†§
Lung US score (score)					
T0	6.0 (1.0)†	6.0 (1.0)†	6.0 (0.0)†	3.0 (1.8)*	1.5 (2.0)*†
T3	6.0 (3.0)	6.0 (3.0)†‡	3.5 (3.8)‡§	3.0 (2.8)	2.0 (2.5)*

Data are expressed as median (interquartile range).

Significant within-group differences are denoted by *P* < 0.05.

Intergroup analysis:

*Significant difference compared to the unassisted group (positive control).

†Significant difference compared to the MV group (negative control).

‡ Significant difference comparing CPAP and HFNO groups.

Time-dependent analysis:

§Significant changes compared to T0.

Abbreviations: HFNO, high-flow nasal oxygen; CPAP, Continuous positive airway pressure; MV, mechanical ventilation; PaO_2_, partial pressure of oxygen in arterial blood; PaCO_2_, partial pressure of carbon dioxide in arterial blood; US, ultrasound.

### Advanced monitoring

CPAP resulted in lower DPes and PRP than the Unassisted and HFNO groups at the end of the study (both *p* < 0.05).

At T0, the Unassisted group had a higher sEMG expiratory effort than the other ones, and the HFNO also presented a higher effort than the MV group (all *p* < 0.05). At T3, MV and CPAP groups had lower effort than the Unassisted (all *p* < 0.05).

**Fig. 1:**
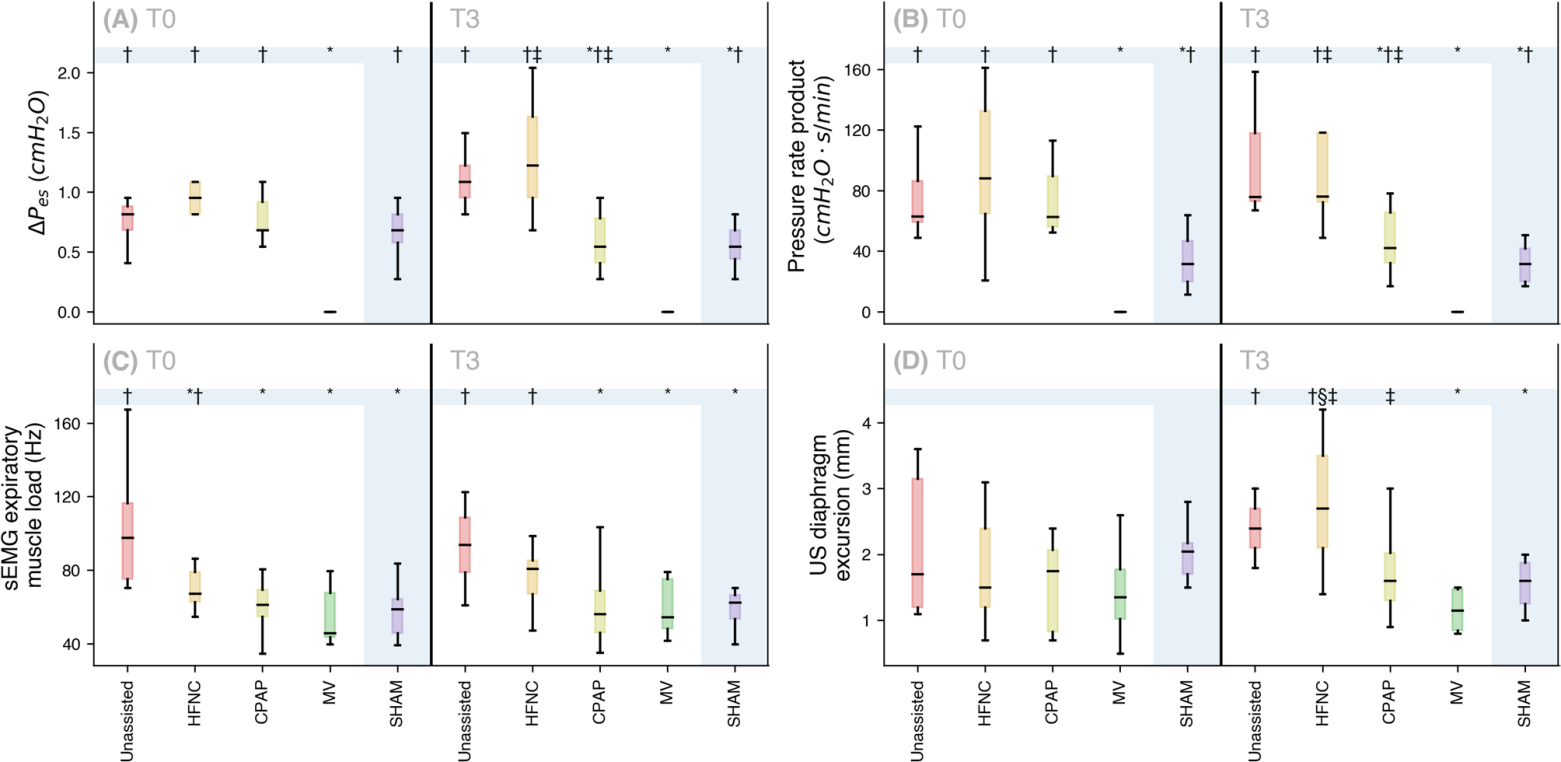
Inspiratory effort, work of breathing, expiratory effort, and diaphragm excursion for the experimental groups at the beginning and end of the observation period. Data are expressed as median (interquartile range). Significant within-group differences are denoted by P < 0.05. Intergroup analysis: *Significant difference compared to the Unassisted group (positive control). †Significant difference compared to the MV group (negative control). ‡Significant difference comparing CPAP and HFNO groups. Time-dependent analysis: §Significant changes compared to T0. Abbreviations: HFNO, high-flow nasal oxygen; CPAP, Continuous positive airway pressure; MV, mechanical ventilation; ΔPes, swing of esophageal pressure; sEMG, surface electromyography; US, ultrasound.

In the intergroup analysis, CPAP had lower diaphragm excursion than HFNO at T3; in the intragroup analysis, HFNO showed a rise in diaphragm excursion (both *p* < 0.05) (Fig. 1).

### Plasma biomarkers

Only MV resulted in lower plasma chemokine growth-regulated protein alpha (GRO-α) levels than the Unassisted group (*p* < 0.05). There were no other differences in any other analyte between groups. An additional file shows plasma biomarker levels [see Additional Table S2].

### Lung injury

**Fig. 2:**
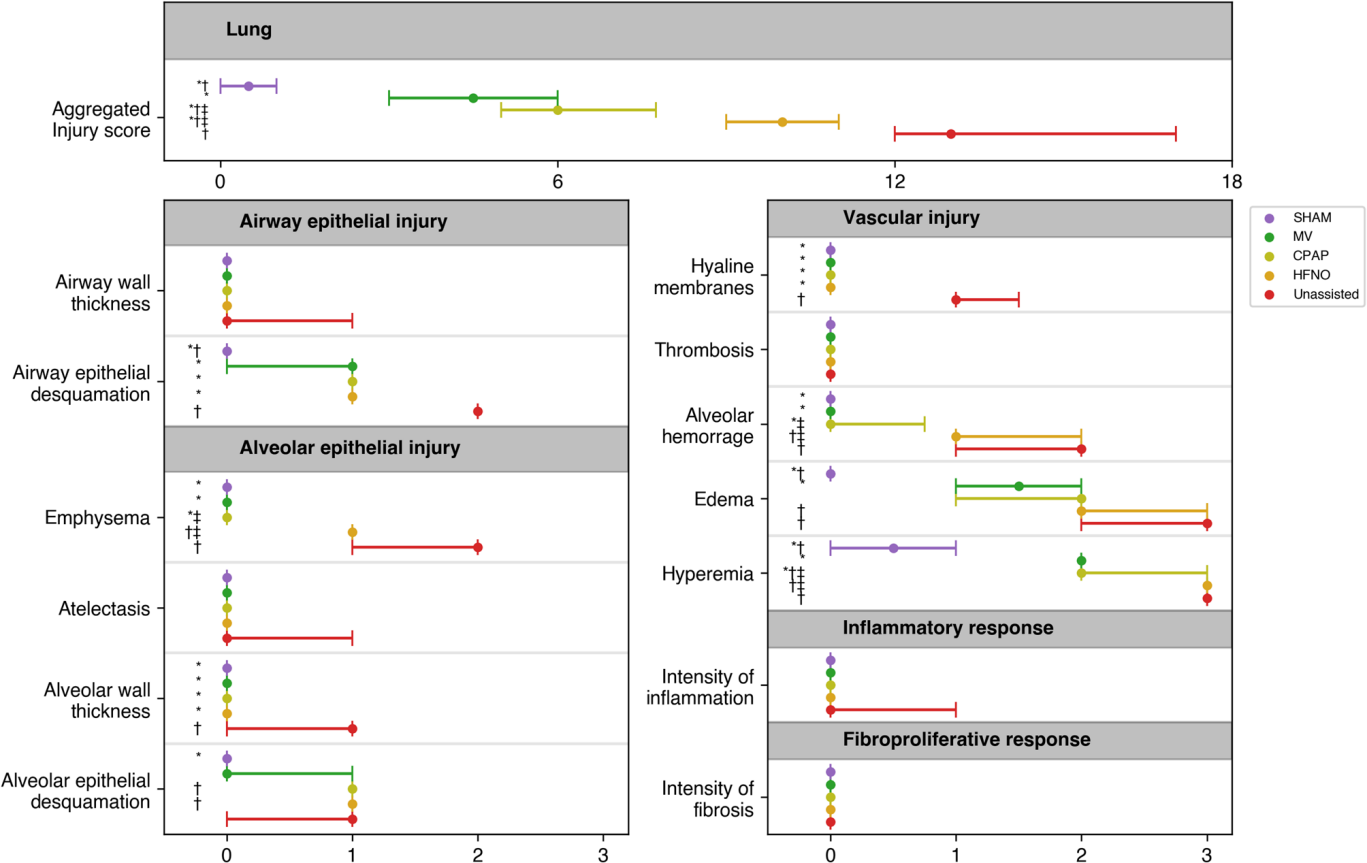
Aggregated semiquantitative lung injury score and its specific components for the experimental groups. Data are expressed as median (interquartile range). Significant within-group differences are denoted by P < 0.05. Intergroup analysis: *Significant difference compared to the Unassisted group (positive control). †Significant difference compared to the MV group (negative control). ‡Significant difference comparing CPAP and HFNO groups. Abbreviations: HFNO, high-flow nasal oxygen; CPAP, Continuous positive airway pressure; MV, mechanical ventilation.

The MV, CPAP, and HFNO groups had a lower aggregated lung injury score than the Unassisted. Injury was mildest in MV, followed by CPAP, and more severe in HFNO (all *p* < 0.05). Compared to the Unassisted group, the MV, CPAP, and HFNO groups had lower airway and alveolar wall thickness, airway epithelial desquamation, and hyaline membranes (all *p* < 0.05). The MV and CPAP groups also presented lower emphysema, alveolar hemorrhage, and hyperemia (all *p* < 0.05). Solely MV showed lower edema (*p* < 0.05). No fibrosis was identified in any subject in any experimental group (Fig. 2).

### Inspiratory obligatory muscle injury (Diaphragm)

In contrast with the Unassisted, the MV, CPAP, and HFNO groups had lower aggregated diaphragm injury scores (all *p* < 0.05). Specifically, all these groups had lower microhemorrhage and necrosis (all *p* < 0.05). CPAP showed a smaller aggregated injury score than MV and HFNO, with no differences between the latter two (both *p* < 0.05). Compared with the HFNO, the CPAP group exhibited lower necrosis (*p* < 0.05).

### Inspiratory accessory muscle injury

The MV, CPAP, and HFNO groups had lower aggregated muscle injury scores in external intercostals and sternocleidomastoid than the Unassisted group. Injury was mildest in MV, followed by CPAP, and more severe in HFNO (all *p* < 0.05).

(a) Intrathoracic inspiratory accessory muscles (external intercostals): the MV and CPAP groups specifically had lower hyperemia, edema, and muscle fiber fragmentation than the Unassisted (all *p* < 0.05). Only the MV group presented lower microhemorrhage (*p* < 0.05).
(b) Extrathoracic inspiratory accessory muscle (sternocleidomastoid): In comparison to the Unassisted group, the MV, CPAP, and HFNO had lower hyperemia, edema, swelling of muscle fibers, and fragmentation (all *p* < 0.05). The MV group also exhibited lower microhemorrhage and nuclei misposition (both *p* < 0.05).

### Expiratory accessory muscle injury

3. (a) Intrathoracic expiratory accessory muscle (internal intercostals): Contrasted to the Unassisted, only the MV group presented a lower aggregated muscle injury score (*p* < 0.05). Specifically, this group presented lower hyperemia and condensation (both *p* < 0.05).
4. (b) Extrathoracic expiratory accessory muscle (rectus abdominis): The MV, CPAP, and HFNO groups showed lower aggregated muscle injury scores than the Unassisted. When quantifying the injury, the MV group presented the lowest, followed equally by CPAP and HFNO (all *p* < 0.05). In particular, the MV, CPAP, and HFNO groups had lower swelling of muscle fibers (*p* < 0.05). Furthermore, the MV group exhibited lower hyperemia, edema, fragmentation, and condensation (all *p* < 0.05). Additionally, the HFNO group presented lower edema and fragmentation (both *p* < 0.05).

We identified minimal or no presence of thrombosis, inflammation, and fibrosis in the diaphragm and accessory respiratory muscles in all experimental groups (data not shown).

**Fig. 3:**
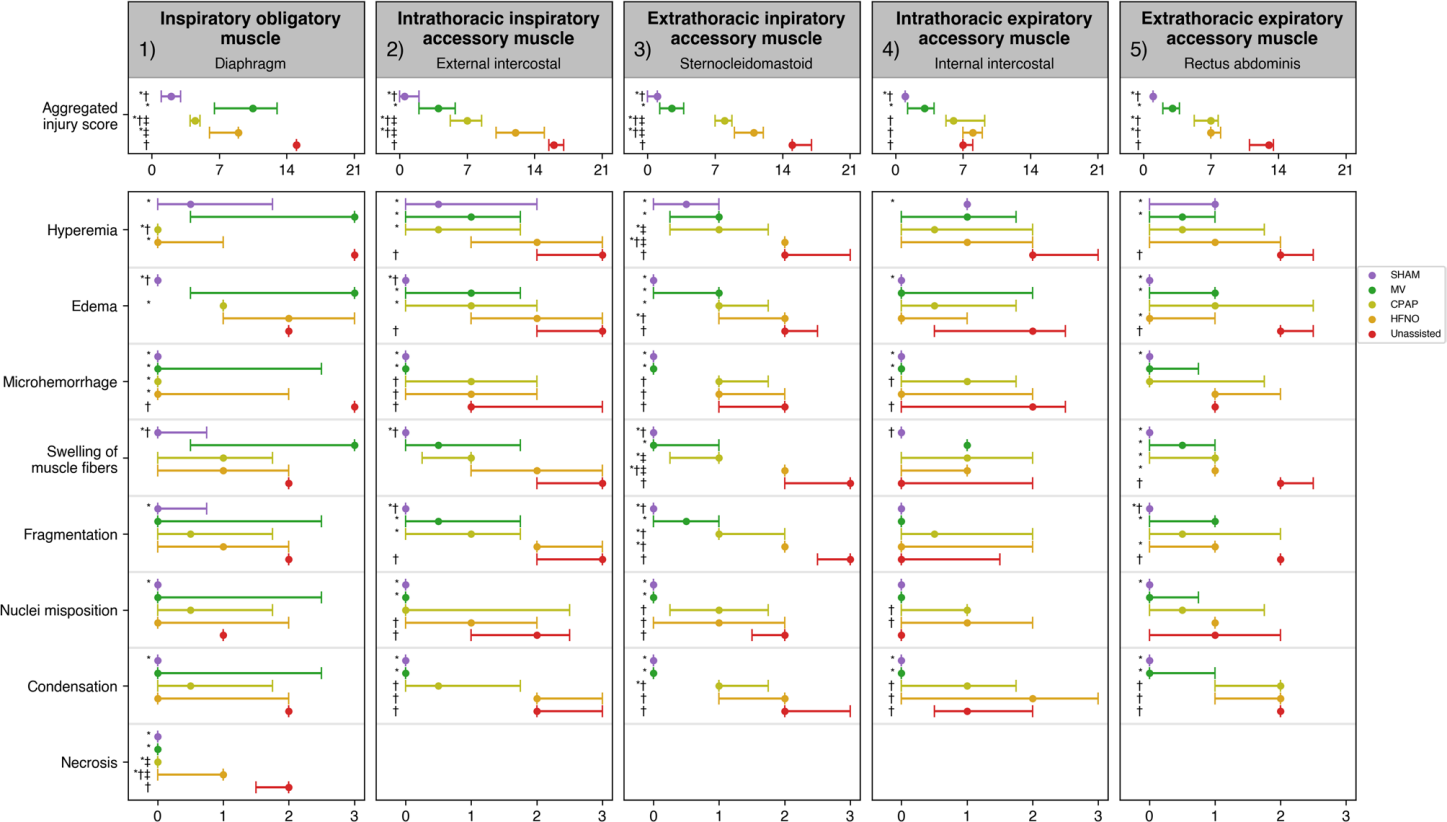
Aggregated semiquantitative respiratory muscle injury scores and their specific components for the experimental groups. Data are expressed as median (interquartile range). Significant within-group differences are denoted by P < 0.05.Intergroup analysis: *Significant difference compared to the Unassisted group (positive control). †Significant difference compared to the MV group (negative control). ‡Significant difference comparing CPAP and HFNO groups. Abbreviations: HFNO, high-flow nasal oxygen; CPAP, Continuous positive airway pressure; MV, mechanical ventilation

**Fig. 4:**
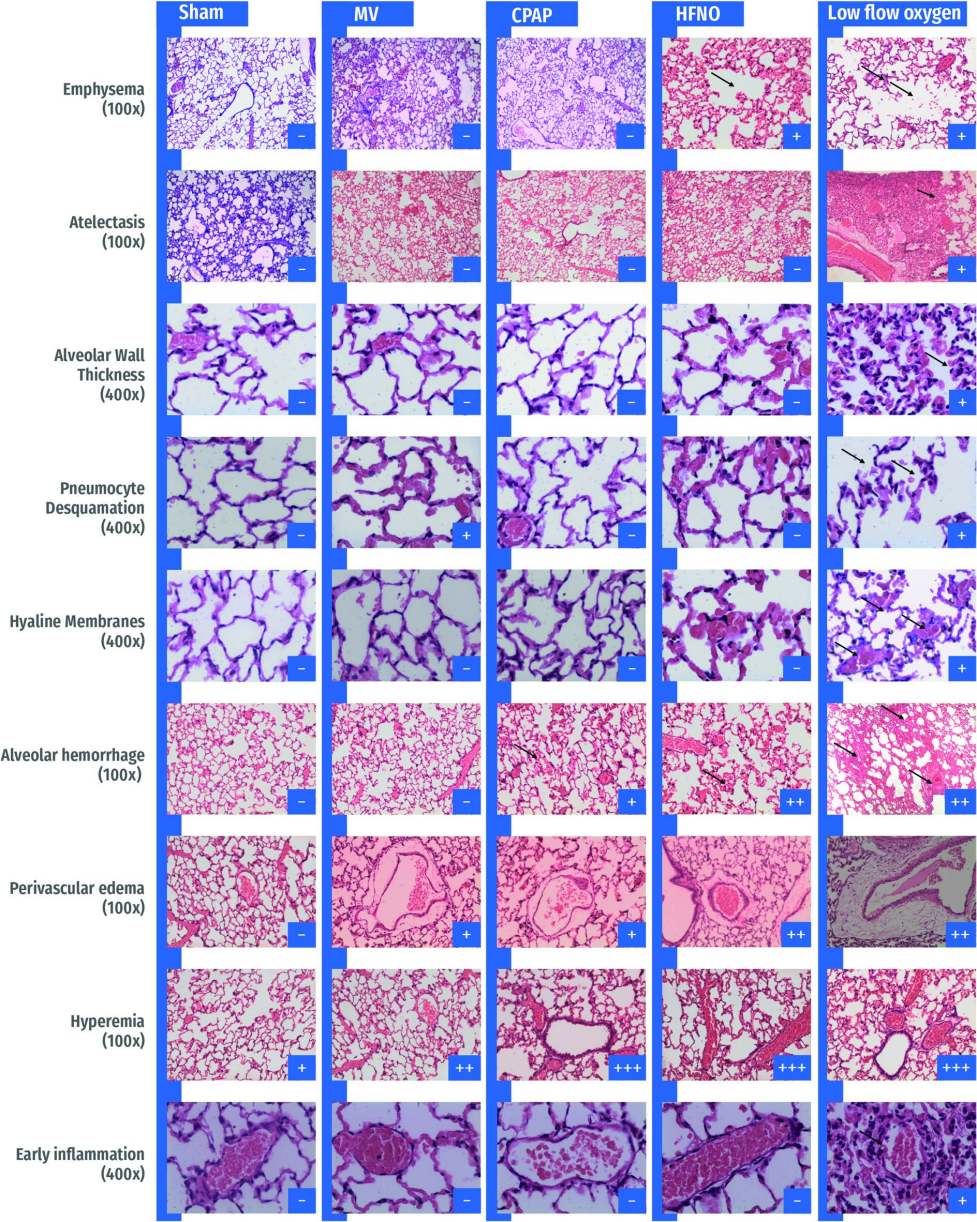
Representative histological sections of lungs from each experimental group, stained with hematoxylin and eosin (H&E). Emphysema (100×): Alveolar rupture was observed exclusively in the HFNO and Unassisted groups. Atelectasis (100×): Detected only in the Unassisted group, characterized by focal areas of complete alveolar collapse. Alveolar wall thickening (400×): Present solely in the Unassisted group, apparently associated with regions of alveolar contraction and early collapse. Pneumocyte desquamation (400×): Observed in the Unassisted group and associated with emphysematous areas and alveolar rupture. Hyaline membranes (400×): Occasionally identified in the Unassisted group. Alveolar hemorrhage (100×): Mild in the CPAP group and moderate in both HFNO and unassisted groups. Perivascular edema (100×): Present in all experimental groups except Sham, with greater severity in the Unassisted and HFNO groups. Hyperemia (100×): Observed across all groups, with markedly increased intensity in the HFNO and Unassisted groups. Early inflammatory changes (400×): Characterized by leukocyte margination along the vascular endothelium and perivascular infiltration, detected exclusively in the Unassisted group. Abbreviations: MV, mechanical ventilation; CPAP, continuous positive airway pressure; HFNO, high-flow nasal oxygen.

**Fig. 5:**
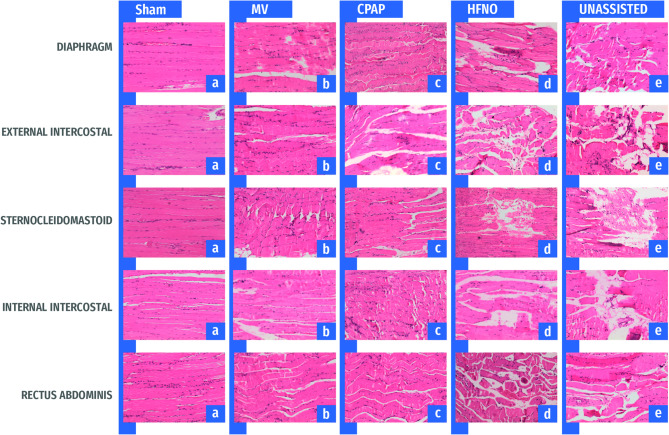
Representative histological sections of respiratory muscles from each experimental group, stained with hematoxylin and eosin (H&E). In contrast with the Unassisted, the MV, CPAP, and HFNO groups had lower diaphragm and accessory muscle injuries. CPAP showed a smaller diaphragm injury than MV and HFNO. Accessory muscle injury was mildest in MV, followed by CPAP, and more severe in HFNO. The Sham group (**a**) showed fiber bundles arranged parallel to each other and no degenerative changes. MV (**b**) and CPAP (**c**) groups exhibited fiber changes such as tumefaction, waviness, and disruption of varying severity. Meanwhile, HFNO (**d**) and Unassisted (**e**) groups displayed more severe fiber fragmentation, along with degenerative changes including sarcoplasm condensation, nuclear pyknosis, internalization, and clustering. Abbreviations: MV, mechanical ventilation; CPAP, continuous positive airway pressure; HFNO, high-flow nasal oxygen.

Figure 3 shows the aggregated and specific injury scores for all respiratory muscles. Representative images of lung and muscle histology for each study group are shown in Figs. 4 and 5.

### Correlation analysis

At T3, there were moderate correlations between DPes and PRP with lung injury (rho = 0.55 and rho = 0.58, respectively, both *p* < 0.001), sternocleidomastoid muscle injury (rho = 0.56 and rho = 0.55, respectively, both *p* < 0.001), and external intercostal muscle injury (rho = 0.56 and rho = 0.58, respectively, both *p* < 0.001). There was no correlation between DPes and PRP with diaphragm injury.

We also found mild-to-moderate correlations between sEMG expiratory effort with lung injury (rho = 0.49 and *p* < 0.001), diaphragm injury (rho = 0.36 and *p* = 0.01), and rectus abdominis muscle injury (rho = 0.47 and *p* < 0.001).

## Discussion

In a three-hour moderate to severe P-SILI model, we studied the functional and structural shielding effects of several respiratory support therapies. Our main findings were as follows: (1) Controlled MV and CPAP improved respiratory compensatory symptoms, ventilation, inspiratory effort, WOB, expiratory effort, and diaphragm excursion; CPAP reduced RR and the loss of lung aeration, and prolonged expiration over time; (2) All respiratory support therapies decreased lung, diaphragm, and accessory inspiratory and expiratory muscle injuries, but with relevant nuances; (3) There was a stepped lung protection: strongest in MV, followed by CPAP, and weakest in HFNO, presenting direct correlations with inspiratory and expiratory effort; (4) Regarding diaphragmatic myotrauma, CPAP was more protective than MV and HFNO. We also found significant correlations with expiratory (but not inspiratory) effort; (5) The protection against inspiratory accessory myotrauma was strongest in MV, followed by CPAP, and weakest in HFNO. MV was more protective against myotrauma in expiratory accessory muscles than CPAP and HFNO. There were direct correlations between inspiratory and expiratory effort with accessory inspiratory and expiratory myotrauma, respectively; (6) These changes occurred despite the lack of differences in oxygenation; (7) Only the MV group resulted in attenuated plasma biomarkers.

In our experimental model, the morphological changes induced by the first hit (surfactant depletion) were displayed in the negative control group (MV). Any additional damage represents a lung and muscle injury propagation pattern, which was markedly greater in our positive control group (standard oxygen therapy)^4,8–10^. This constituted the second hit, triggered by static and dynamic mechanobiological mechanisms. In other words, this group allowed us to characterize P-SILI. The main study groups were CPAP and HFNO, and the primary outcome was lung and respiratory myotrauma. The several physiological assessments performed allowed us to better understand the underlying mechanisms.

P-SILI occurs predominantly on the vascular side of the blood-gas barrier^4^, probably caused by large cyclic blood flow oscillations resulting from inspiratory negative pressure swings and active expiration in vulnerable lungs. If the position and geometry of the diaphragm are disadvantageous enough to affect its contraction and relaxation kinetics, it worsens^8,30^. P-SILI is triggered by both the concentric and eccentric contractions of obligatory and accessory inspiratory muscles and due to active expiration through functional recruitment of accessory expiratory muscles^31^. In line with these mechanisms, we observed direct correlations between lung injury and inspiratory/expiratory respiratory efforts. However, the effort-related second hit also occurred on the epithelial side of the barrier^4^, regionally amplified by stress raisers, evidenced by high lung regional strain and heterogeneity^9^, tidal recruitment^8^, strain rate^8^, and the pendelluft phenomenon^32^. Also, the higher RR with a shorter expiratory time, avoiding full deflation, resulting in dynamic air trapping, could promote P-SILI^8^. Controlled MV prevents the mechanobiological phenomena and lung injury progression in the preclinical but also in the clinical setting^1–3,9,10^. In acute hypoxemic respiratory failure (AHRF) patients, the transition from spontaneous to controlled MV resulted in a shift toward ventral and more homogeneous ventilation, an improvement in oxygenation, and a drop in systemic inflammation^33^. Accordingly, elevated respiratory drive and inspiratory effort, and increased driving pressure during assisted ventilation have been related to lower survival and prolonged ventilation^34–36^.

Protective effects on P-SILI were present in both noninvasive support therapies, specifically in epithelial and vascular injuries. Effects of noninvasive respiratory support therapies on respiratory effort and oxygenation are widely known. These therapies are commonly used in mild to moderate ARDS, preventing MV in up to two-thirds of them^12,37–40^, and therapy success is associated with improved survival^41,42^. However, failure is related to poor outcomes^30^, raising concerns linked to the concept of P-SILI.

In our current study, the benefits of CPAP seem to be attributable to pulmonary and non-pulmonary mechanisms. The improvement in lung aeration suggests the reversal of lung collapse, in accordance with lung recruitment in a surfactant depletion model. But PEEP also repositions the diaphragm in its original location^29^, preserving its geometry^8^, improving its contraction and relaxation kinetics^8^, and allowing a safer contraction^4,8,30,43^.

HFNO slightly reduced lung injury and under-assistance myotrauma, despite no decrease in inspiratory and expiratory effort nor enhanced aeration was observed for this group. Shielding effects could be explained by the addition of PEEP, which allowed diaphragm repositioning. The Hering-Breuer reflex may have influenced the heterogeneity in the physiological response to HFNO therapy^44^. Furthermore, the severity of primary lung injury may have prevented a greater effectiveness.

Our study also morphologically characterized myotrauma in obligatory and accessory muscles. While all support therapies were found to be protective, CPAP was the best in shielding the diaphragm, and MV was the most efficient in protecting accessory inspiratory and expiratory muscles. Several mechanisms related to under- and over-assistance myotrauma have been identified, such as strong concentric^6,45^ and eccentric contractions^46^, excessive unloading^47^, and disproportionately higher PEEP^48^. The greater the inspiratory and expiratory unloading of the accessory muscles, the lower the myotrauma, a relationship strongly aligned with the concept of under-assistance myotrauma. However, maintaining diaphragm activity, as promoted by CPAP, was the most protective strategy, suggesting that excessive discharge is also a relevant injury mechanism (over-assistance myotrauma under controlled MV)^31^. In accordance, a lower gene expression of markers of diaphragm inflammation and proteolysis was reported in a study in rats with mild lung injury under pressure-support compared to pressure-controlled ventilation, despite having the same dynamic transpulmonary driving pressure^49^. Surprisingly, we found that diaphragmatic injury was correlated with expiratory but not inspiratory effort. So, the concurrent active expiration may promote diaphragmatic myotrauma. Accordingly, a recent experimental study reported that CPAP reduced the velocity of relaxation across the entire diaphragm curvature and prevented myofibrillar damage^4,8^.

Controlled MV was the only support therapy that prevented biotrauma. This could be explained by the greater tissue protection induced by MV, as well as by methodological factors, such as the low sensitivity of plasma protein biomarkers in a short-term model. Accordingly, a brief P-SILI model showed increased expression of genes related to inflammation and pathological mechanotransduction in regions of high regional strain^7^. Other methodological factors that may also have attenuated the systemic inflammatory response were the model itself (surfactant depletion induces mechanical stress rather than inflammation)^50^ and the use of specific pathogen-free animals.

Our study has several limitations: (1) Although the DPes values seem quite low compared to human studies, they are similar to those reported in other murine studies, probably determined by the distensibility of the chest wall^22,51]– [52^. (2) The lack of a rodent-specific esophageal balloon allowed us to only measure DPes in a non-blind way and without the possibility of excluding esophageal elastance^53^. In turn, it also did not allow us to quantify PEEP during HFNO. (3) Despite being a dependent variable, hypercapnia likely attenuated the possible additional protective effects of HFNO; an increase in respiratory drive induces greater respiratory effort and tissue damage. In a clinical setting, hypercapnic acidosis may result in a higher incidence of HFNO failure^54^. (4) We did not measure regional 3D lung strain and diaphragm deformation maps, which are reference methods to quantify mechanobiological phenomena related to respiratory effort in a preclinical setting^8,9^. Operational restrictions prevented the use of µCT due to the short time of the experimental phase and the wide variety of tools used in this study. (5) Anesthetic drugs could modify the respiratory pattern and may affect the effectiveness of noninvasive respiratory support therapies, especially if the collapse of upper airways occurs. (6) Due to technological limitations, we were unable to evaluate physiological dead space. This could have been a relevant pathophysiological mechanism in our model because the instrumental dead space varied between the interfaces used in different groups. (7) The small number of subjects might lead to type II error. (8) Finally, caution should be exercised when extrapolating experimental findings to the clinical setting.

Despite these limitations, our study highlights the direct relationship between the decrease of inspiratory and expiratory effort by respiratory support therapies and protection against P-SILI and *myotrauma* of the diaphragm and accessory respiratory muscles in experimental lung injury. Furthermore, the functional changes detected in real time through simple and advanced monitoring confirm a tissue correlation in lungs and respiratory muscles, in agreement with P-SILI and under- and over-assistance myotrauma concepts.

## Supplementary Information

Below is the link to the electronic supplementary material.

Supplementary Material 1

## Data Availability

The datasets used and analyzed during the current study are available from the corresponding author on reasonable request.
