# Supplementary material for: CPAP and high-flow nasal oxygen also reduce lung, diaphragm, and accessory muscle injury in experimental self-inflicted lung injury
Source: Sci Rep. 2026 Feb 11;16:8399. doi: 10.1038/s41598-026-39564-7 (PMC12972071; doi:10.1038/s41598-026-39564-7)

**SUPPLEMENTARY MATERIAL**

**CPAP and high-flow nasal oxygen also reduce lung, diaphragm, and accessory muscle injury in experimental self-inflicted lung injury**

Sonia Reveco^1^, Felipe M. Llancalahuen^2^, Paola Caviedes^1^, Andrés Silva^3^, Javier Contreras^4^, Carlos González^5^, Benjamín Erranz^6^, Agustín Pérez^6,7^, Juan P. Cruces^8^, Daniel E. Hurtado^6,7^, Pablo Cruces^1,8^.

^1^Unidad de Paciente Crítico Pediátrico, Hospital El Carmen Dr. Luis Valentín Ferrada, Santiago, Chile.

^2^Laboratory of Translational Research in Critical Care (LTRCC), Departamento de Medicina Intensiva, Facultad de Medicina, Pontificia Universidad Católica de Chile, Santiago, Chile.

^3^Laboratorio de Genética y Patogénesis Bacteriana, Centro de Investigación de Resiliencia a Pandemias, Facultad de Ciencias de la Vida, Universidad Andres Bello, Santiago, Chile.

^4^Laboratorio integrativo de Biomecánica y Fisiología del Esfuerzo (LIBFE), Escuela de Kinesiología,^,^ Universidad de los Andes, Santiago, Chile.

^5^School of Veterinary Medicine, One Health Institute, Faculty of Life Sciences, Universidad Andres Bello, Santiago, Chile.

^6^Department of Structural and Geotechnical Engineering, School of Engineering, Pontificia Universidad Católica de Chile, Santiago, Chile.

^7^Institute for Biological and Medical Engineering, Schools of Engineering, Medicine and Biological Sciences, Pontificia Universidad Católica de Chile, Santiago, Chile.

^8^Laboratory of Translational Research in Critical Care, Center for Research on Pandemic Resilience, Faculty of Life Sciences, Universidad Andres Bello, Santiago, Chile.

**Corresponding Author:** Pablo Cruces, Unidad de Paciente Crítico Pediátrico, Hospital El Carmen Dr. Luis Valentín Ferrada, Santiago, Chile. Phone: +56 2 26120641, e-mail: [pcrucesr@gmail.com](mailto:pcrucesr@gmail.com)

**Table S1.** Physiological data for the experimental groups at baseline, after saline lavage.

|  | Unassisted | HFNO | CPAP | MV | Sham |
| --- | --- | --- | --- | --- | --- |
| **Weight (g)** | | | | | |
|  | 320 (17.5) | 320 (35) | 318 (30) | 322 (30) | 320 (23) |
| **Sex (Male/Female)** | | | | | |
|  | 5/5 | 5/6 | 5/5 | 5/5 | 5/5 |
| **SpO2 (%)** | | | | | |
|  | 85.0 (2.5) | 84.0 (2.0) | 81.0 (3.8) | 83.0 (1.5) | 100.0 (1.5)*† |
| **RR (breaths/min)** | | | | | |
|  | 103.0 (30.0) | 98.0 (34.0) | 92.0 (18.0) | 90.0 (0.0) | 46.0 (24.5)*† |

Data are expressed as median (interquartile range).

Significant within-group differences are denoted by P < 0.05.

Intergroup analysis:

*Significant difference compared to the unassisted group (positive control).

†Significant difference compared to the MV group (negative control).

Abbreviations: HFNO, high-flow nasal oxygen; CPAP, Continuous positive airway pressure; MV, mechanical ventilation; SpO_2_, oxygen saturation as measured by pulse oximetry; RR, respiratory rate.

**Table S2.** Plasma biomarkers for the experimental groups at the end of the study.

|  | Unassisted | HFNO | CPAP | MV | Sham |
| --- | --- | --- | --- | --- | --- |
| **Inflammatory cytokines** | | | | | |
| IL-1β (pg/mL) | 28.9 (78.1) | 6.4 (61.0) | 44.3 (55.1) | 7.4 (29.7) | 7.9 (29.4) |
| TNF-α (pg/mL) | 3.2 (5.0) | 0.0 (3.2) | 3.8 (5.8) | 0.0 (2.4) | 0.0 (3.2) |
| **Immunomodulatory cytokine** | | | | | |
| IL-2 (pg/mL) | 0.5 (3.2) | 0.0 (1.1) | 0.6 (4.3) | 0.0 (0.0) | 0.0 (0.0) |
| **Chemokine** | | | | | |
| GRO-α (pg/mL) | 58.5 (65.8)† | 21.4 (39.2) | 16.1 (9.6) | 13.2 (11.0)* | 17.4 (57.7) |
| **Cell adhesion molecules** | | | | | |
| ICAM-1 (pg/mL) | 1016 (598) | 853 (155) | 1154 (355) | 716 (493) | 797 (439) |
| VCAM-1(pg/mL) | 1189 (86) | 1254 (155) | 1260 (260) | 1083 (384) | 1198 (296) |
| **Cellular damage markers** | | | | | |
| Total CK (U/L) | 52.3 (13.9) | 39.9 (11.3) | 41.6 (9.6) | 52.2 (16.4) | 41.6 (9.6) |
| LDH (U/L) | 359 (220) | 437 (155) | 271 (155) | 525 (383) | 238 (92) |

Data are expressed as median (interquartile range).

Significant within-group differences are denoted by P < 0.05.

Intergroup analysis:

*Significant difference compared to the unassisted group (positive control).

†Significant difference compared to the MV group (negative control).

Abbreviations: HFNO, high-flow nasal oxygen; CPAP, Continuous positive airway pressure; MV, mechanical ventilation; IL-1β, Interleukin-1 beta; TNF-α, Tumor necrosis factor-alpha; IL-2, Interleukin-2, GRO-α, Growth Regulated Protein-alpha; ICAM-1, Intercellular adhesion molecule-1; VCAM-1, Vascular Cell Adhesion Molecule-1; CK, Creatine kinase ; LDH, Lactate dehydrogenase.

**Figure S1.** Method used for esophageal manometry and representative images of the experimental groups.

A) With the subject in the supine position, a short water-filled catheter was inserted into the esophagus, and the proximal lumen was transduced. Its location was confirmed with an “occlusion test”.

B) Inspiratory effort was assessed as negative esophageal pressure swings (ΔPes) on a rodent-specific monitor, recording the maximum negative deflection of the esophageal pressure. Since the monitor auto-scales, ΔPes can be displayed on the Y-axis. In controlled MV, we recorded a ΔPes = 0.

C) Representative images of all the studied groups, showing higher ΔPes in the unassisted and HFNO groups.

Abbreviations: MV, mechanical ventilation; CPAP, Continuous positive airway pressure; HFNO, high-flow nasal oxygen.


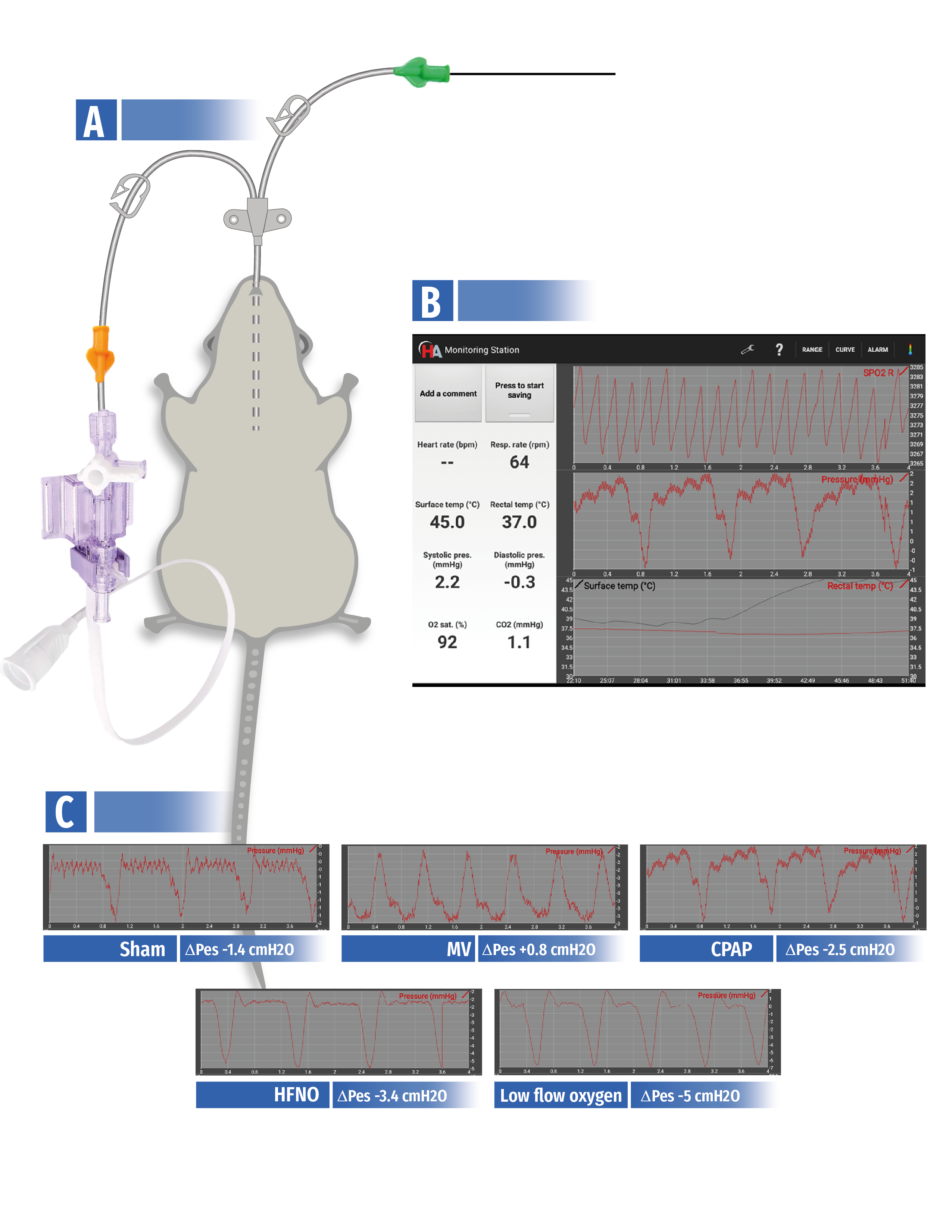


**Figure S2.** Method used for diaphragm excursion and representative images of the experimental groups.

A) Evaluation of diaphragm excursion in the right hemithorax, which reflects the amplitude of the active movement of the diaphragm during the respiratory cycle. The probe was placed at the junction of the anterior and posterior axillary lines and the lower edge of the right costal arch. The liver was used as the acoustic window for the diaphragm. The probe was pointed to the head and back, and diaphragm excursion and inspiratory time were displayed under M-mode.

B) The depth was set to 3cm, and the broadband linear array probe, at 19 MHz. In the top-left corner, *“distance”* represents diaphragm excursion in cm.

C) Representative images of all the studied groups, showing higher diaphragm excursion in the unassisted and HFNO groups.

Abbreviations: MV, mechanical ventilation; CPAP, Continuous positive airway pressure; HFNO, high-flow nasal oxygen.

**
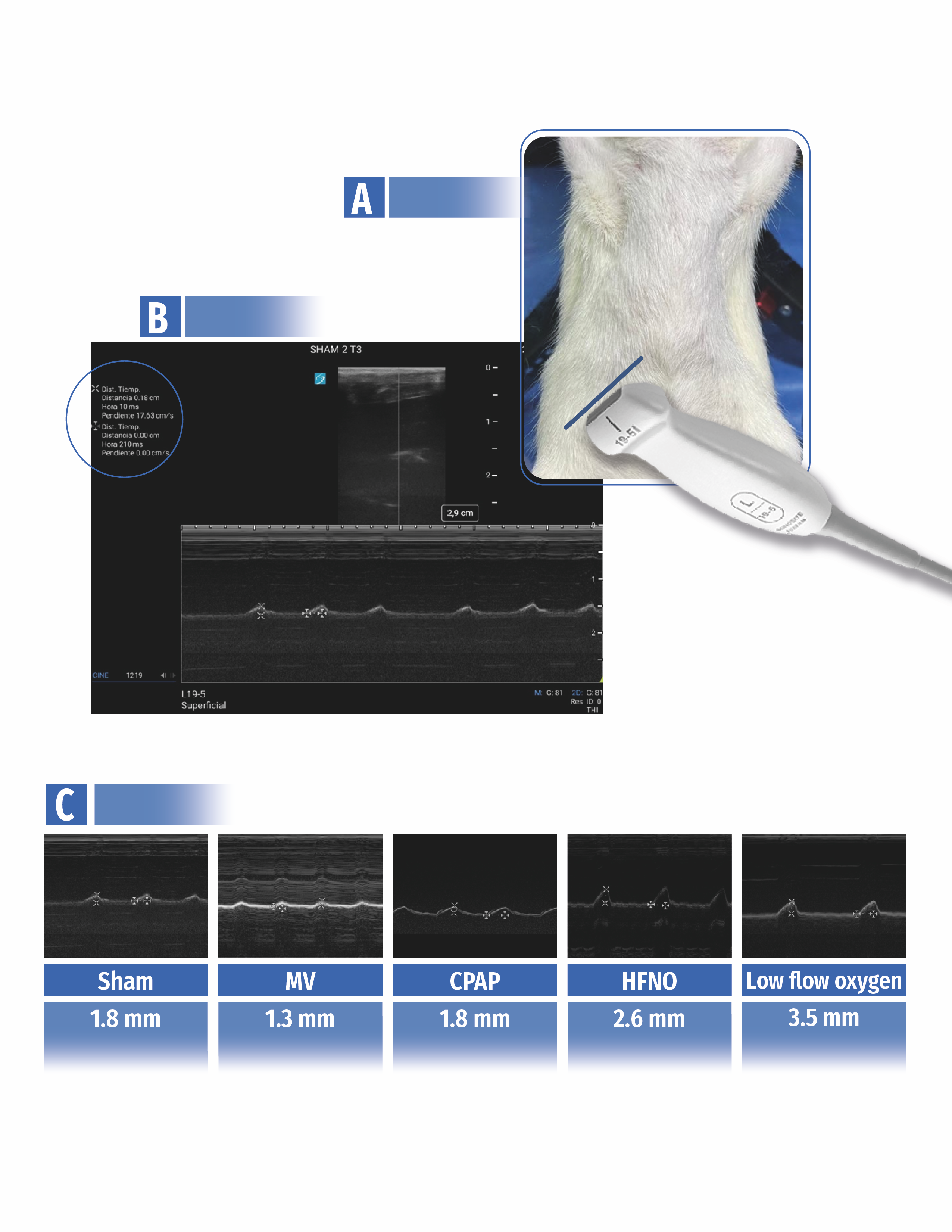
**

**Figure S3.** Method used for lung ultrasound (US) score and representative images.

A) Each lung was divided into four regions of interest: anterior right, lateral right, anterior left, and lateral left. For each zone, a score of 0–3 was assigned according to the observed pattern. The four US patterns and the scores given for each were as follows: 1) Normal pattern, presence of lung sliding and artifactual horizontal A-lines (0 points); 2) B-pattern, presence of two or more well-defined vertical B-lines extending from the pleural line (1 point); 3) Severe B-pattern, multiple confluent vertical B-lines extending from the pleural line (2 points); and 4) Lung consolidation, presence of a tissue structure with or without hyperechoic punctiform images resembling air bronchograms (3 points). The worst finding in each region was used to calculate the lung US score, ranging from 0 to 12 points, by adding the individual scores. Images were obtained using B-mode. Lung US score quantified the loss of lung aeration.

B) The depth was set to 3cm, and the broadband linear array probe was set at 19 MHz.

C) Representative images for each of the scores, ranging from 0 to 3.

D) Representative images of all the studied groups, showing higher loss of lung aeration in the unassisted and HFNO groups.

Abbreviations: MV, mechanical ventilation; CPAP, Continuous positive airway pressure; HFNO, high-flow nasal oxygen.

**
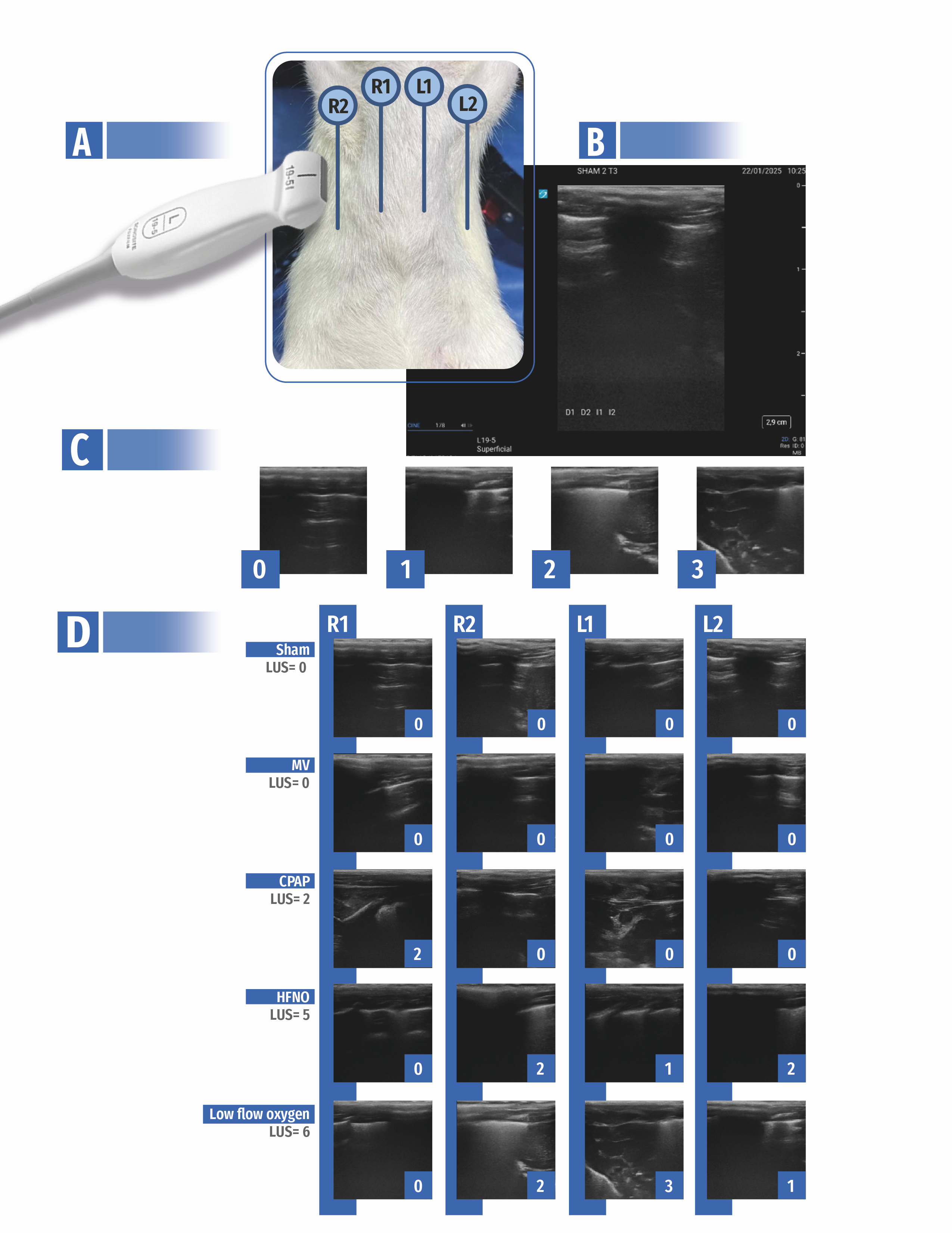
**

**Figure S4.** Method used for surface electromyography in the rectus abdominis muscle and representative images of the experimental groups.

A) Placement of electromyography (EMG) sensors and accelerometers on the target muscle.

B) Delsys EMGworks software interface displaying the frequency spectrum of an EMG signal and the data export option. The figure shows the power spectral density (PSD) of the electromyographic signal from the muscle.

C) Representative images of all the studied groups. Spectral analysis reveals higher power at high frequencies in the unassisted and HFNO groups, indicating greater expiratory effort.

Abbreviations: MV, mechanical ventilation; CPAP, Continuous positive airway pressure; HFNO, high-flow nasal oxygen.

**
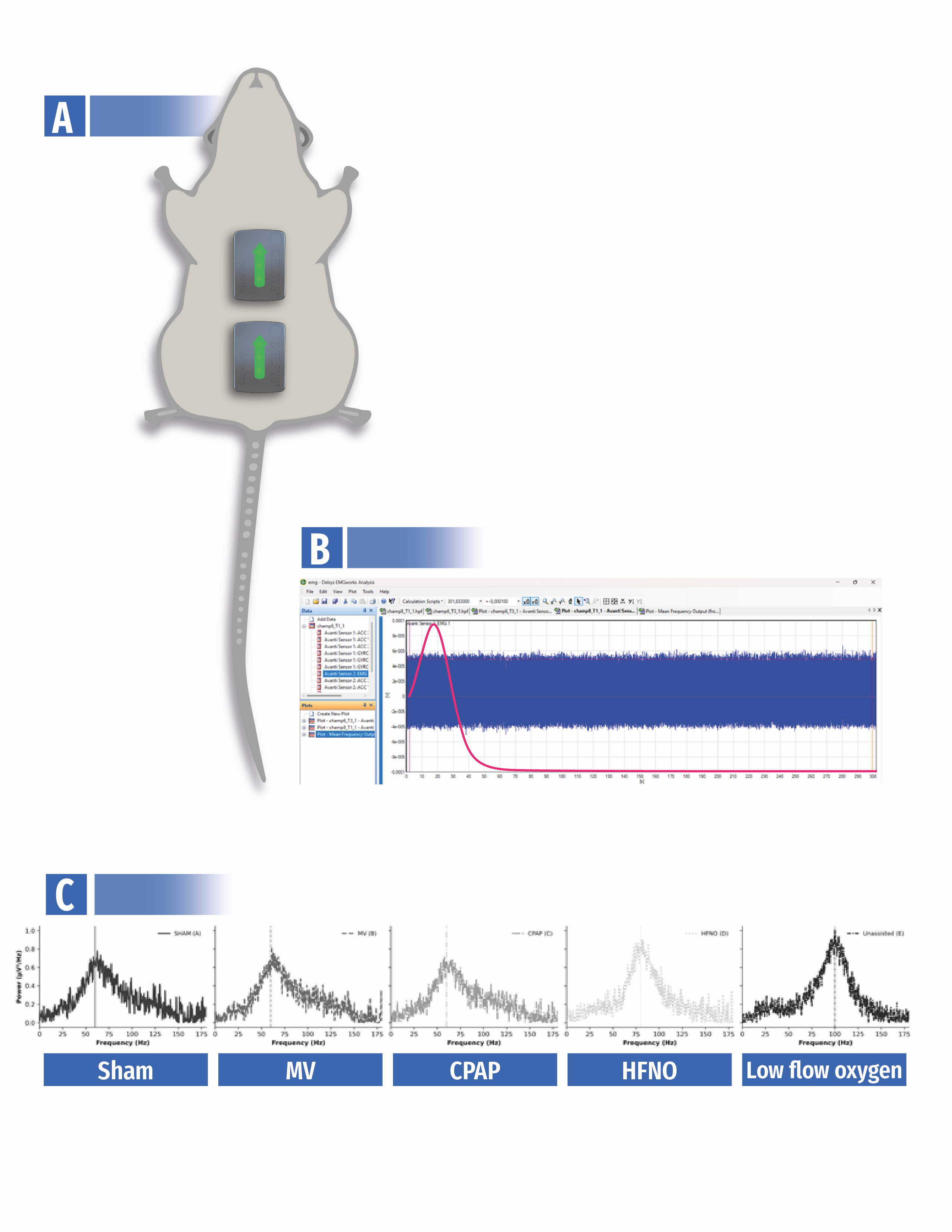
**

**Figure S5.** Schematic flowchart and timeline of the experimental protocol.

Abbreviations: MV, mechanical ventilation; CPAP, Continuous positive airway pressure; HFNO, high-flow nasal oxygen; ABG, arterial blood gases. Data acquisition: esophageal manometry, surface electromyography, ultrasound of the diaphragm and lungs, and ABG.


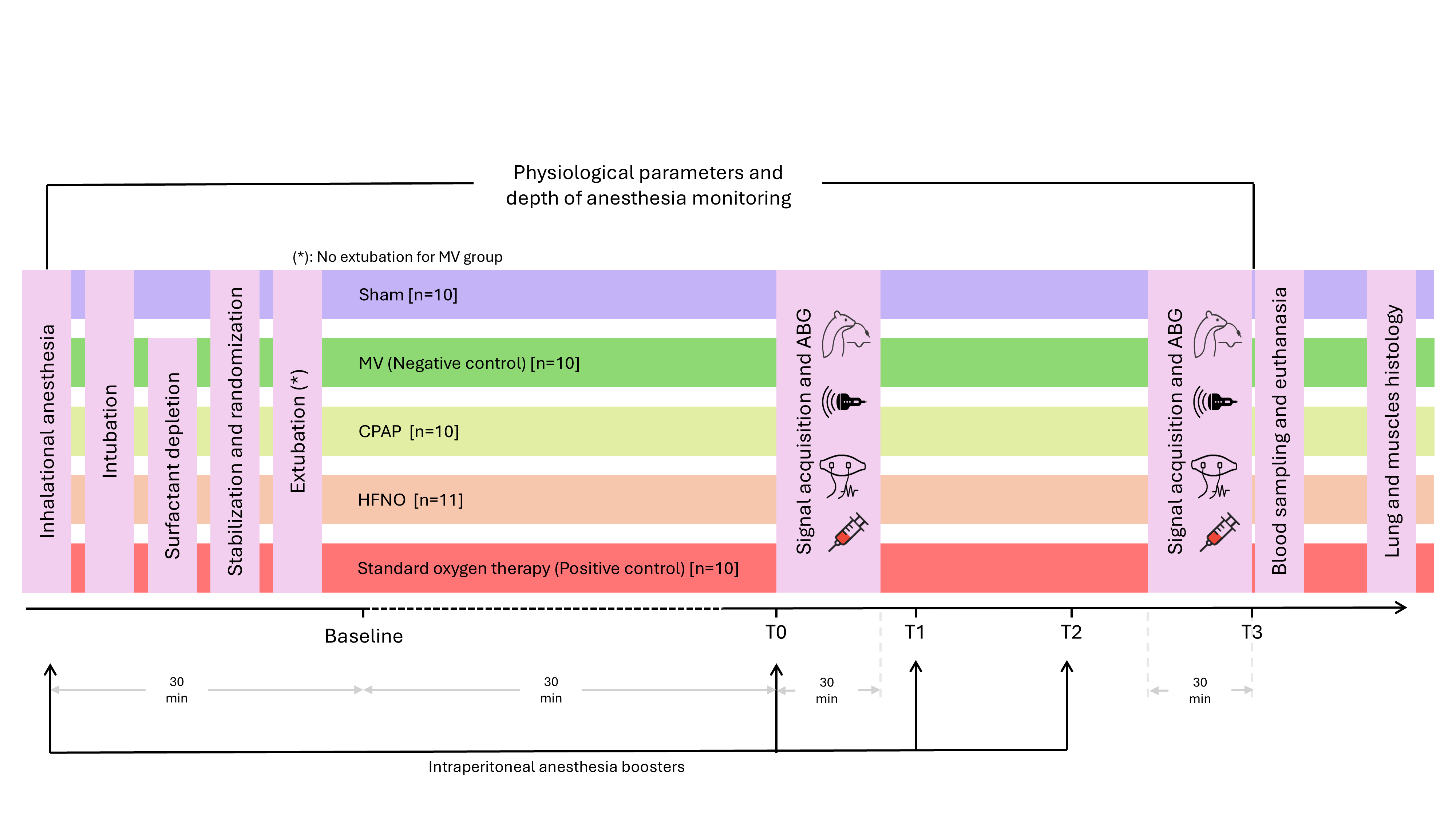

Supplement: Supplementary file 1 — Supplementary Material 1 [file 41598_2026_39564_MOESM1_ESM.docx]
